# Service Life Prediction of Type-IV Composite CNG Cylinder under the Influence of Drivers’ Refueling Habits—A Numerical Study

**DOI:** 10.3390/polym15112480

**Published:** 2023-05-27

**Authors:** Kazem Reza Kashyzadeh, Aleksandr Vyacheslavovich Marusin

**Affiliations:** Department of Transport, Academy of Engineering, RUDN University, 6 Miklukho-Maklaya St., Moscow 117198, Russia

**Keywords:** polymer composite tank, type-IV CNG tank, polyethylene liner, variable wall thickness, driver habits, service life prediction, finite element analysis

## Abstract

The new generation presented for CNG fuel tanks of vehicles (type-IV) is made entirely of composites. The reason for that is to prevent the sudden explosion of metal tanks and to use the advantage of gas leakage in composite materials. Previous research has shown that type-IV CNG fuel tanks also have challenges such as variable wall thickness in outer shell parts, which are prone to failure under cyclic refueling loading. The optimization of this structure is on the agenda of many scholars and automakers, and in this regard, there are many standards for strength assessment. Despite reporting injury events, it seems that another parameter should be included in these calculations. In this article, the authors have attempted to numerically investigate the effect of drivers’ refueling habits on the service life of type-IV CNG fuel tanks. For this purpose, a 34-L CNG tank made of glass/epoxy composite, polyethylene, and Al-7075T6, respectively, for the outer shell parts, liner, and flanges was considered as a case study. Moreover, a real-size measurement-based finite element model validated in the corresponding author’s previous research was used. The loading history was applied as internal pressure according to the standard statement. Furthermore, considering different behavior of drivers for refueling, several loading histories with asymmetric details were applied. Eventually, the results obtained for different cases were compared to experimental data in symmetrical loading. The results showed that, based on the car’s mileage, the driver’s behavior in the refueling process can significantly reduce the service life of the tank (up to 78% of the predicted life based on the standard methodology).

## 1. Introduction

In recent years, researchers and industrialists have sought to find alternative fuels to fossil fuels due to various reasons, such as reducing air pollution or limiting fossil fuel resources, etc. The results of some studies show that adding some substance or nanoparticles to the fuel can improve the performance of the engine or reduce the amount of air pollution (results obtained from the analysis of exhaust fumes). However, the primary basis of these fuels has also been fossil fuels, and it does not help much in view of limited sources of this fuel, although old and experienced automotive manufacturers have also been interested in using gasoline and diesel engines. Unfortunately, this fuel has far less latent energy compared to petrol. In addition, the noise of such engines during operation is more than that of diesel engines. Countries that have natural gas resources have tried to use this potential in their transportation industry. Thus, they have used Compressed Natural Gas (CNG) as a suitable substitute for fossil fuels in cars. After that, electric and hybrid cars have also entered the market. Recently, there has been a lot of research on using hydrogen as a fuel for vehicles. In comparison, all these fuels have disadvantages, limitations, and advantages over each other, which are beyond the scope of this paper. In fact, for the storage of any gas fuel such as CNG or H2, a safe tank is needed because it is exposed to many risks such as bursting, and this issue is directly related to the lives of the driver and passengers. Therefore, the focus of this paper is on gas fuel tanks in cars and their strength.

At first, CNG fuel tanks were made entirely of metal. The first limitation was their large size, which forced them to be placed in the trunk of the car. The second problem was their heavy weight, which led to a solid weight being added as an extra load to the car’s rear suspension system. Since this load was not seen during the initial design of the car and capsules were sometimes manually put on some cars after leaving the factory, many problems were observed for the comfort of the rear passengers of the car, such as in the maneuvers of the car in turns, sometimes the initial acceleration of the car, etc. Apart from these, there were many reports of deaths and financial injuries that were published due to the bursting and explosion of these tanks, which led to people’s distrust in this system. Therefore, new generations of CNG fuel tanks were presented by researchers. In this regard, all-metal tanks are known as the first generation, and all-composite tanks including a polymer liner and an external body made of composite are known as the last generation, i.e., the fourth generation. The biggest advantage of type-IV CNG fuel tanks is their use of the property of gas leakage. In other words, at the time of failure, the gas passes through the various layers of the composite and the gas is sensed (before the tank fully opens and bursts). This is despite the fact that in metal tanks, as soon as the initial crack is created, the crack grows and the gas escapes, an explosion occurs, and the tank breaks into pieces and is thrown around like thousands of shrapnel; each of these pieces can cause the death of a person. In the following, a brief overview of researchers’ achievements in the field of evaluating the strength of composite tanks and their optimization will be discussed.

Gehandler and Lönnermark experimentally studied the behavior of composite CNG tanks exposed to fire [1]. Moreover, Ou et al. conducted a laboratory study of the behavior of tanks exposed to local and extensive fire in an aluminum liner composite tank filled with hydrogen [2]. They reported that both internal gas pressure and temperature parameters do not change much when the tank is in the vicinity of local fire. Moreover, filling medium and tank pressure have weak impact on the activation time of TPRD, but they have a remarkable influence on the activation time of pressure-activated PRD. Hence, it is vital to pay attention to various tank-monitoring techniques and ensure the tank’s health during operation. In this regard, Acoustic Emission Energy (AEE)-based Signal Mapping Methods (SMMs) have been employed to identify the source location of damages on the composite CNG tank caused by external shock [3]. Glisic and Inaudi used long-gauge fiber optic sensors to monitor the health of full composite CNG tanks [4]. To this end, after the tank was fully manufactured, the sensors were wrapped around it. They analyzed monitoring results at several levels, and damage was detected using algorithms that combine overall deformation and changes in tank stiffness. One of the factors that can increase the temperature of the internal gas or the temperature of the tank body is filling it quickly. Saferna et al. discussed the results of thermodynamic analysis of the CNG composite tank due to the fast filling process [5]. They also claimed that the composition of natural gas affects the rapid filling time of the tank and compressor performance due to the filling process. Consequently, this process should be as short as possible, and during this time, the tank should be filled with as much gas as possible. Kim and Choi utilized computer modeling and fractography methods to perform risk analysis of composite tanks for compressed natural gas fuel [6]. They reported that the main cause of failure in CNG composite tanks was the interference and floating effect between the clamp bolts and the tank. They recommended that the tank’s installation components, i.e., clamp belts and bolts, be redesigned and, in addition, new assembly processes be implemented. Moreover, it is also necessary to carry out periodic inspections every three years to check the health of other parts such as valves, pipelines, etc. It should be noted that conducting only experimental research is very expensive and time consuming. In addition, in this case, it is not possible to manufacture a lot of tanks and perform various tests on a real scale without achieving the correct design. On the other hand, with the development of simulation software and their very good accuracy in examining the behavior of various materials under different working conditions, it is logical to make a sample and perform various tests after obtaining the most optimal state, or the final design. In this regard, Kim et al. have evaluated the structural safety of CNG composite tanks based on Finite Element (FE) simulation [7]. To this end, they used the von Mises yield criterion and Tsai-Hill theory. In addition, FE simulation has been used to study the behavior of CNG tanks made of composite considering different twist angles [8]. The researchers used Kevlar fiber for the outer body and finally stated that considering the twist angle of 35 degrees, the least deformation can be achieved in the tank. However, they only considered the constant thickness for the tank wall, which is different from reality. Nouri et al. studied experimentally and numerically the performance of CNG composite tanks under static load and considering variable wall thickness [9]. For the first time, they used the Finite Element Model (FEM) based on experimental measurements in this field, and by conducting stress analysis, they identified the critical area prone to failure. After that, they used different static failure criteria for composite materials to obtain fracture strength and calculate safety factors in each of the composite layers in the critical region. Finally, they stated that the most accurate criterion should include 3D normal and shear stress components. Seyedi et al. optimized the type-IV composite tank under internal pressure loading according to conditions beyond the working conditions and testing conditions stated in the standard, in other words, the endurance limit until the tank explosion [10]. They modeled the composite tank as 23 layers in such a way that the first layer was considered as a liner (7.5 mm), the next 10 layers were thick glass fibers (0.9 mm), and the next 12 layers were thinner than glass fibers (0.75 mm). They benefited from the Design of Experiments (DOE) technique to perform the response surface analysis; the pressure created in the critical zone and the tank deformation were considered as outputs. Eventually, they showed that compared to metal tanks, lightweight composite tanks are more resistant to internal pressures, resulting in a 30% reduction in the weight of composite tanks and a 20% reduction in deformation under working pressure. Nouri et al. attempted to obtain the optimum fiber twist angle in the composite CNG tank in two cases of constant and variable wall thickness under static loading conditions [11]. They reported that considering the lowest value of von Mises stress as the objective function, the most optimal angle is equal to 23 and 15 degrees for the composite tank with constant and variable thickness, respectively. Debondue investigated the advantages of glass fiber applications in the construction of CNG composite tanks, and the efficiency of such tanks was assessed [12]. A hybrid FE-RSM method has been presented to optimize fatigue lifetime of a polymer composite CNG tank [13]. Using the results of the finite element simulation and its coupling to the analysis of response surface method, the researchers were able to find the optimal values for the wall thickness in different parts of the tank, including the main body in the form of a cylinder and the beginning and end of the tank in the shape of a hemisphere, as well as the connection area of the tank to the aluminum flanges. In addition, they also optimized the fiber twist angle. They reported that the fatigue life of the tank manufactured using the optimal values is 2.4 times longer than that of the tank in the initial state. Recently, the accuracy of predicting the service life of the composite CNG tank has been evaluated by employing different fatigue failure criteria [14]. Despite all the efforts made by various researchers and industrialists in the field of strength assessment of vehicle fuel tanks and their structural optimization, there are still the problems of tank failures. Hence, it seems that in addition to the investigated cases, another parameter influences the service life of the tanks, which has been overlooked by designers. In this regard, the authors believe that the driver’s behavior in refueling can be one of these factors. Therefore, in this article, the authors for the first time discussed the behavior of drivers in refueling the gas tank on the service time of the tanks by numerical simulation. To this end, dynamic analyses were performed using the validated FEM of a composite tank with variable wall thickness. Different loadings were considered based on the capacity of the tank, and finally, the service life of the tank was reported according to the distance traveled.

## 2. Methodology

**Figure 1:**
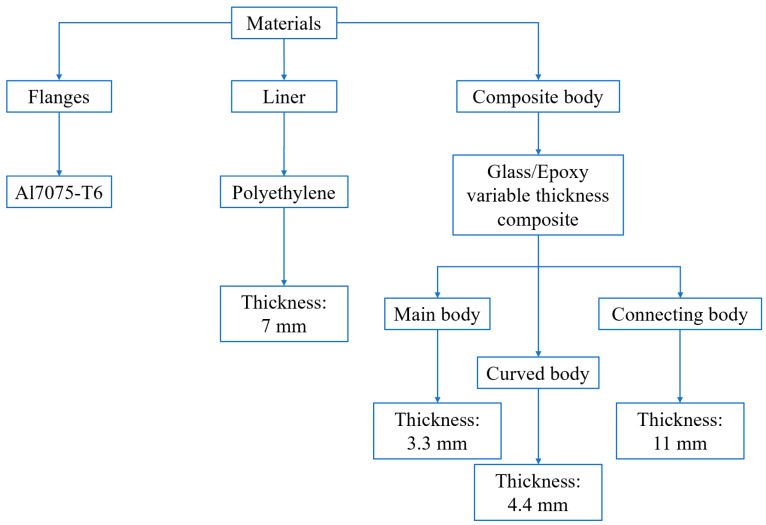

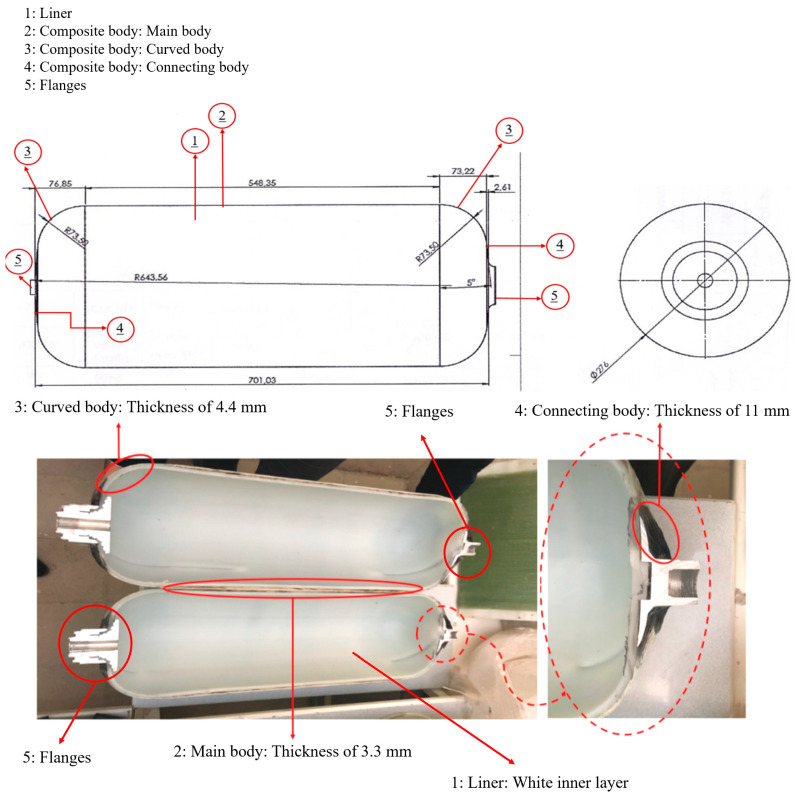
Schematic of the shape and size of the 34-L composite tank and the thickness of its different walls based on the experimental measurements.

In this research, a type-IV composite CNG tank (made in a company affiliated with the Iranian automobile industry) with a capacity of 34 L for use in a passenger car was studied. From the viewpoint of material, this tank contains three main parts called the liner (inner shell of the tank), the composite body (outer shell of the tank), and the beginning and end flanges on both sides of the tank. In this case, the material of the flanges is 7000 series aluminum with T6 heat treatment. The liner is made of polyethylene with a constant and uniform thickness of 7 mm. However, the composite body has variable thicknesses. To facilitate the analysis, the composite shell part is divided into three categories, including main body (i.e., the cylindrical part), the curved body (i.e., the hemisphere parts at the beginning and end of the tank), and the connecting body between the tank and the flanges, with each part having the same thickness. In other words, from one part to another part of the composite shell, the thickness changes significantly. This division is based on the experimental measurements that the first author made in his previous studies [11,13,14]. Figure 1 illustrates a schematic of the dimensions of the tank and also shows the thickness of its different parts.

**Figure 2:**
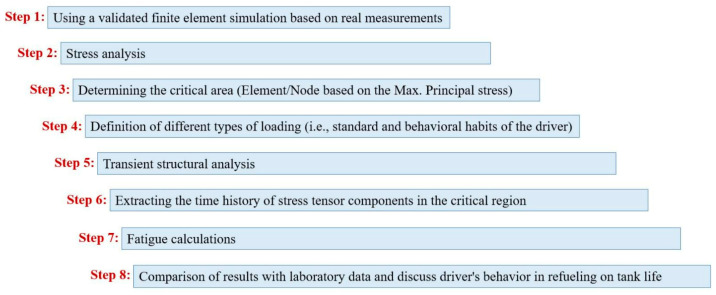
Step-by-step workflow in the present study.

According to the manufacturer’s report, the fiber of Glass 2400, Epoxy resin UN3082, and UN 2735 hardener are used to create the composite shell. Moreover, the volume ratio of the fiber to composite is 50% and the thickness of all layers is 0.55 mm. Furthermore, the fiber twist angle is 17 degrees. To perform the analysis in this study, the validated FEM was used. More detailed information about this FEM can be found by referring to ref. [13], which is based on experimental measurements obtained from cutting the constructed tank in the Iranian automotive industry. Figure 2 displays the workflow of this research.

Based on this workflow, the first step is to simulate the finite element of the tank according to the shape and size, define the connections and adhesion of different parts to each other, define various materials in the software, i.e., ANSYS WORKBENCH 2016, Ansys, Canonsburg, PA, USA, and assign the suitable material behavior for each part. In this regard, Step 1 has been carried out in previous studies, and in the present research, the validated FE simulation was used [11,13,14]. The second step is dedicated to stress analysis under maximum internal pressure loading according to the value declared in the tank strength measurement standard. This analysis is done statically, and its result is reported in the next step (i.e., Step 3) as the detection of the critical area and the element/node prone to failure based on the criterion of the maximum principal stress. In the fourth step, the various loadings are defined based on the behavioral habits of the driver in the refueling process. Afterward, transient dynamic analysis is performed under the loading history obtained in the previous step for two cycles of refueling and emptying the tank. The results of this analysis include extracting the time histories of the stress tensor components ($\left[\sigma_{ij}\right]\mathrm{MPa}$) in the critical element/node. In Step 7, fatigue calculations are performed by employing a fatigue failure criterion for composite materials. Since these results are preliminary research and need further study in future research, therefore, in this study, it has been tried to use the simplest criterion in order to reduce the computational costs and their complexities, as well as the need for material coefficients that should be obtained only from axial fatigue tests. Finally, in the last step, the results are compared with experimental data, and the achievements of this research are discussed.

## 3. Numerical Analysis

**Figure 3:**
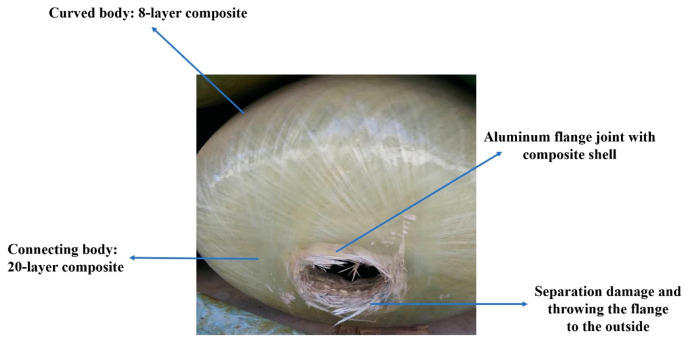
Tank failure location in the static test until bursting [11].

**Figure 4:**
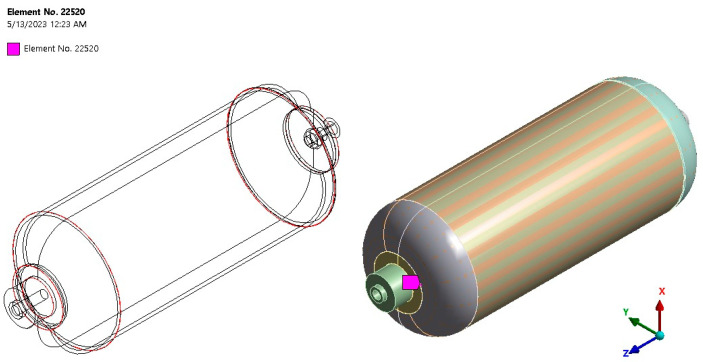
The exact location determined as the prone area for fatigue failure in the tank, i.e., node No. 22520.

In previous studies, it has been stated that the results of FE analysis under the maximum internal loading stated in the tank strength testing standards (i.e., the minimum and the maximum internal pressure of 20 and 200 bar, respectively) indicate that the absolute principal stress (S = 206.32 MPa) occurs in the connecting body, which is also consistent with the static test data of the tank [13]. In addition, in other research, it was reported that during the instantaneous strength test of the tank, the flange was dislodged and thrown out quickly [11,14]. Figure 3 illustrates the failure location of the tank in the strength test under static load until bursting [11]. Accordingly, in the current research, it was assumed that the critical zone, which is susceptible to failure due to cyclic loading, is the connecting body made of glass/epoxy composite with 20 layers and related to the node No. 22520. Figure 4 demonstrates the exact location of this node in the tank. Hence, the fatigue calculations are performed only in this location because the fatigue analysis for the whole system is very time consuming, and on the other hand, other parts are not as severely under the risk of failure like this location.

**Figure 5:**
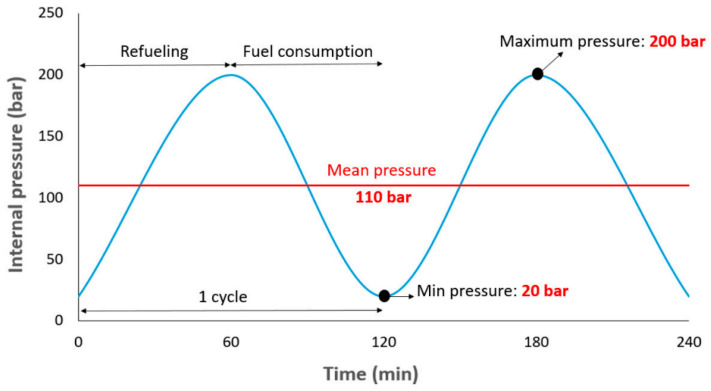
Standard loading details considered for dynamic analysis and fatigue life prediction of CNG fuel tank [13].

For fatigue analysis, five different loading modes were considered. One of the modes is Standard Loading (SL) [15], which has been considered by scholars in previous studies. The standard loading along with the details of refueling cycles and fuel consumption is shown in Figure 5. For the other loading modes, it was assumed that the driver refuels before the vehicle runs out of the fuel. In other words, the status of the CNG fuel in the car is indicated by small lights on the display screen in front of the steering wheel. Thus, usually this monitor has 5 green lamps and if the CNG fuel tank is full, 5 green lamps are on, and if the CNG fuel tank is empty, all the lamps are off. Therefore, in standard loading mode as shown in Figure 5, the driver refuels when the number of green lamps is zero. Hereupon, four other modes are considered for status when the number of green lamps during refueling is equal to 1, 2, 3, and 4. Table 1 reports the characteristics of cyclic loading in different modes investigated in this research. Remember that in the nomenclature, DB stands for driver behavior, and the number after it indicates the number of green lights on the car’s fuel status monitor.

The first author, in a previous article [14], investigated the accuracy of various failure criteria for composite materials under fatigue loading in tanks considering standard loading. Nouri et al. reported that some criteria are not appropriate, and compared to the laboratory results, they lead to an error of over 40%. Moreover, some of the criteria show an error of less than 20%. In this regard, the most accurate criterion was Epaarachchi and Clausen with a 15% error, but due to complex calculations and the need for many material constants, the best criterion for predicting the fatigue life of composite tanks with variable-thickness wall was the Fawaz–Ellyin criterion, which has good accuracy. In fact, this criterion is able to predict the fatigue life of the tank according to the specifications provided by the manufacturer as well as the standard loading, with an error of about 17% compared to the experimental data. On the other hand, this criterion includes the effects of both normal and shear stresses, simultaneously. Therefore, this criterion was used in the present study. It proposed a semi-logarithmic linear relationship between the applied cyclic stress (S) and the number of cycles to failure (N) as follows [16]:

(1) $$S=m\times \log\left(N\right)+b$$

where S and N are applied cyclic stress and number of cycles to failure corresponding to S, respectively. Further, m and b are defined as material coefficients whose values depend on both material properties and loading conditions. In addition, this criterion believes that there is a reference line (S−log⁡(N)) for the fatigue behavior of composite materials. Therefore, Equation (2) represents this reference line [16].

(2) $$S_{r}=m_{r}\times \log\left(N\right)+b_{r}$$

Further, the relationship between the material coefficients obtained from the laboratory results of the fatigue test and this reference line is as follows [16]:

(3) $$m=f\left(a_{1},a_{2},\theta \right).g\left(R\right).m_{r}$$

(4) $$b=f\left(a_{1},a_{2},\theta \right).b_{r}$$

where $a_{1}=\frac{\sigma_{y}}{\sigma_{x}}$ and $a_{2}=\frac{\tau_{xy}}{\sigma_{x}}$ are the first and second biaxial ratios, respectively, R is the stress ratio, and θ represents the fiber orientation. Considering the above equations, this model can be generalized as follows:

(5) $$S\left(a_{1},a_{2},\theta ,R,N\right)=f(a_{1},a_{2},\theta )\left[g\left(R\right)m_{r}\log\left(N\right)+b_{r}\right]$$

In addition, parameters of m and b are based on a line of general S − log(N) so that this model can estimate them for any θ, R, a_1_, and a_2_.

## 4. Results and Discussion

**Figure 6:**
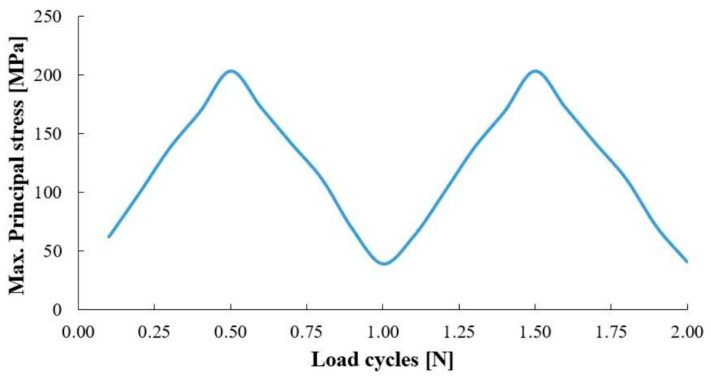
The history of the maximum principal stress as a result of the stress analysis under two cycles of standard loading.

**Figure 7:**
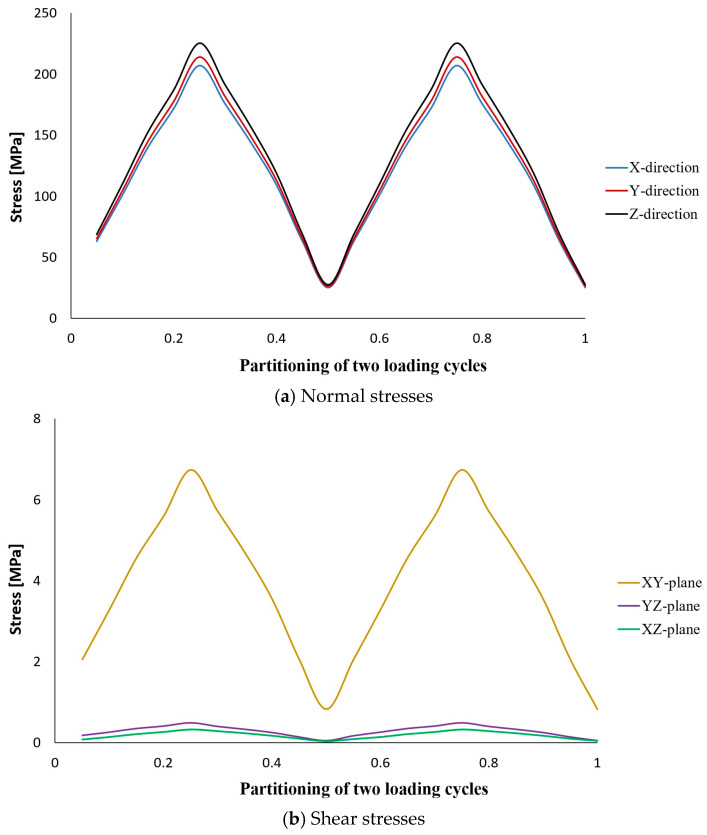
Stress tensor components at node No. 22520 caused by two cycles of loading according to the standard load characteristics.

**Figure 8:**
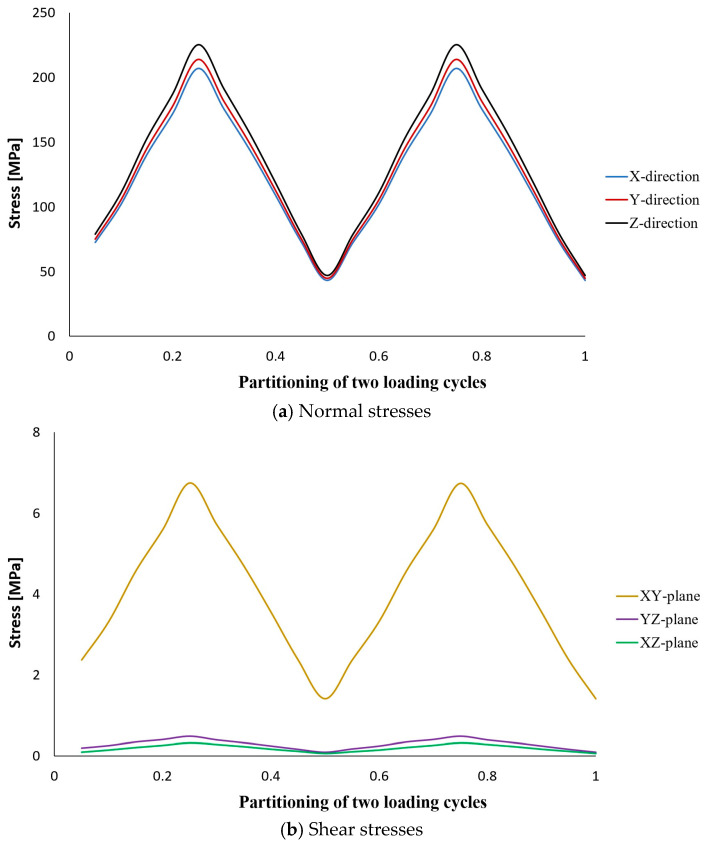
Stress tensor components at node No. 22520 caused by two cycles of loading according to the DB-1 characteristics.

**Figure 9:**
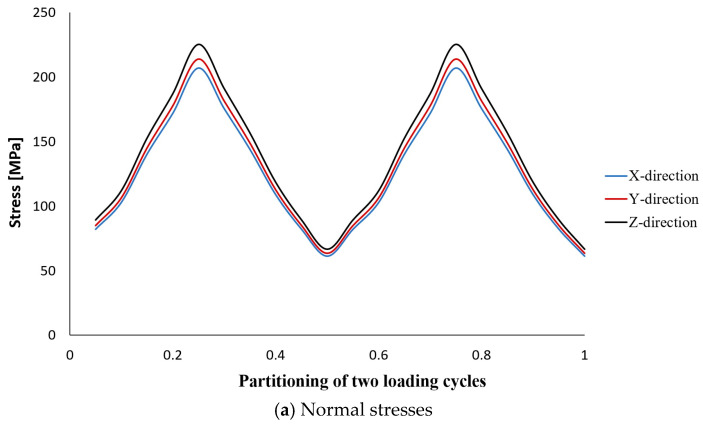

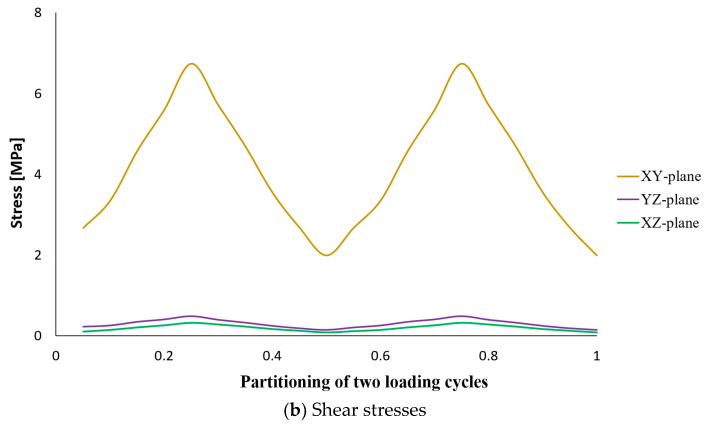
Stress tensor components at node No. 22520 caused by two cycles of loading according to the DB-2 characteristics.

**Figure 10:**
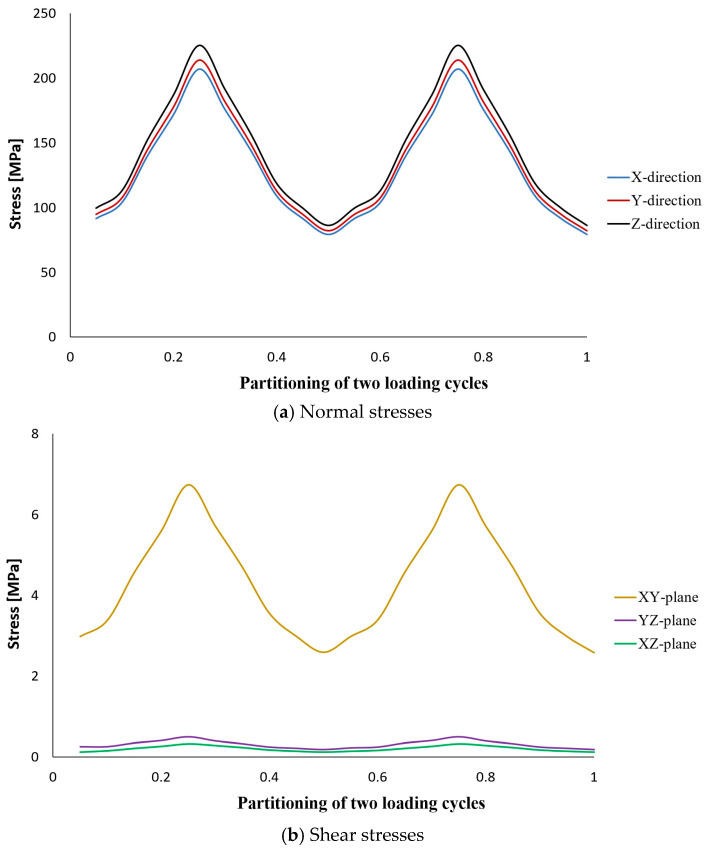
Stress tensor components at node No. 22520 caused by two cycles of loading according to the DB-3 characteristics.

**Figure 11:**
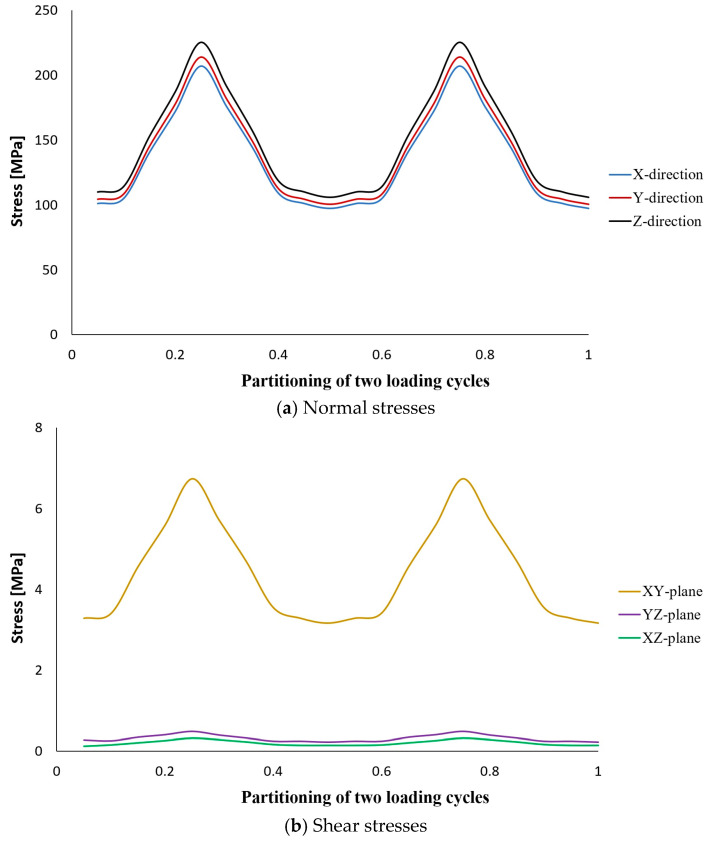
Stress tensor components at node No. 22520 caused by two cycles of loading according to the DB-4 characteristics.

The results of stress analysis and extracting the history of maximum principal stress in the whole tank are in accordance with the results presented in previous reports [11,13,14]. As is clear from the diagram shown in Figure 6, its maximum value in one cycle loading is about 206 MPa. Moreover, this stress has occurred in the same place of failure presented earlier, i.e., the interface between the composite layer and the aluminum flange (in the connecting body and close to the flange). Both of these cases indicate that the previously validated model was used well. Next, different transient dynamic analyses were performed for different loadings according to the specifications presented in Table 1. It should be noted that all the analyses were performed for two loading cycles. The histories of the stress components at the critical node, i.e., 22520, were extracted and displayed separately in Figure 7, Figure 8, Figure 9, Figure 10 and Figure 11.

**Figure 12:**
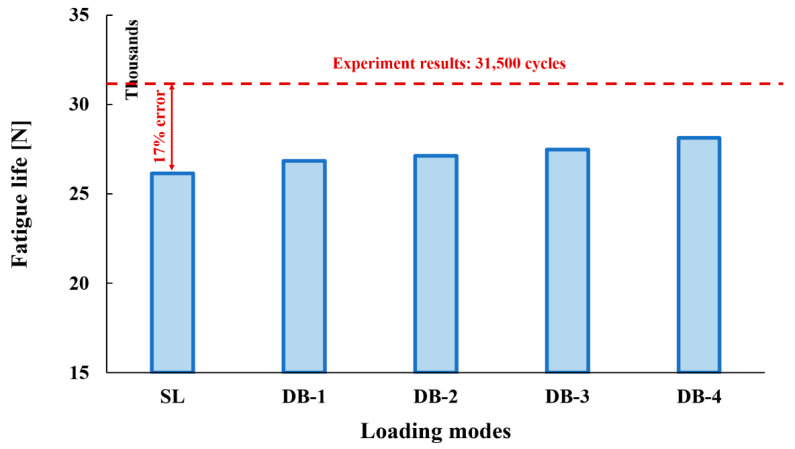
Fatigue life prediction in the critical node of the composite CNG tank considering different loading modes, including the standard and driver behavior effects.

The obtained results for all loading modes show that the shear stress in the XY-plane (as shown in Figure 4, the circular cross-section of the cylinder is in the XY-plane and the length of the cylinder is in the Z-direction. In this case, the layering thickness of the composite shell is in the Y-direction.) has a much higher value compared to the other two shear components. This achievement is also consistent with the statements in other articles [13,14]. Furthermore, fatigue life at the critical node for different loading modes, including SL, DB-1, DB-2, DB-3, and DB4, was calculated by using the stress values and employing the Fawaz–Ellyin criterion. Figure 12 presents the fatigue results.

The results indicate that the lowest fatigue life is when the considered loading mode is in accordance with the standard loading. In other words, the worst conditions are presented for calculating the strength of tanks. This means that the behavioral habits of the driver in refueling do not have a negative effect on the fatigue life of the tank. On the other hand, the biggest changes obtained in comparison with the standard loading mode are related to DB-4, which has a 7.6% difference. It is true that according to the behavior of the driver in the refueling process, the load ratio changes in the fatigue cycles and reaches from 0.1 in the standard mode to 0.46 in the DB-4 mode. However, the fatigue life is increased; in this way, the mean stress during loading has increased (from 110 to 146 MPa), but due to the constant maximum load when the tank is filled, i.e., 200 MPa, the range of stress changes in loading cycles is reduced. Therefore, this loading can be assumed as a superposition of two types of static and dynamic loading at the same time. The mean and constant stress is statically applied to the tank permanently. Now, with the increase in the loading ratio, the range of dynamic loading changes, which is the main cause of fatigue damage, decreases. Therefore, damage occurs later in the material, and the life of the system increases. However, the whole story does not end with these reports. Adequate attention should be paid to the fact that, in this study, a tank with a capacity of 34 L was used. The fuel consumption of the desired car is 6 L per 100 km; therefore, one can drive about 166 km with a full CNG tank. According to the obtained fatigue life values, which show the number of refueling and emptying cycles of the tank, in standard mode, this car can travel near 4,340,000 km before the tank breaks, which is more than 15 years of car operation; but when refueling is carried out when the tank is not empty, the loading cycle has not covered 166 km. For example, when a refueling process is carried out in DB-4 mode, instead of 166 km, the previous tank has traveled 33.2 km because the driver has refueled and started the second loading cycle by only turning off a green light from the fuel checker monitor. Hence, the mileage of the car before the failure of the tank is around 934,000 km. This value is reduced by about 79% compared to the standard mode. Table 2 illustrates a summary of the service life of the composite CNG tank via the mileage of the vehicle. In addition, it has been observed many times in daily life that when the CNG tank is empty and refueling, the temperature of the tank body does not change appreciably. Meanwhile, if refueling is carried out when the tank is not empty, the body of the tank is also heated to such an extent that it cannot be touched in some cases. This issue, that is, thermal shocks, can also affect the life of the CNG tank, which the authors will address in future studies.

## 5. Conclusions

In the present study, the authors have tried to investigate the effect of drivers’ behavior habits in the refueling process on the service life of a type-IV composite CNG tank. In addition to considering the standard loading mode, four other loading modes were also considered according to the number of lamps in the vehicle’s fuel controller monitor. In addition, in order to estimate the fatigue life, a simple criterion for failure of composite materials was used (due to the reduction in complexity of calculations and the subsequent low computational cost and less need for the material coefficients that must be obtained from the experiments) because this research examines an initial hypothesis and will be a basis for future research. The most important achievements of this study are the following:

1. An increase in the load ratio assuming the maximum loading is constant does not have a negative effect on the fatigue life of the tank, and assuming the mean stress is static and constant, the range of dynamic loading changes is reduced.
2. Based on the fatigue calculations and the number of loading cycles to failure for the composite material, it can be concluded that the worst condition, the lowest fatigue life, is related to the condition mentioned in the tank strength measurement standard.
3. If the service life of the CNG tank is considered based on the mileage of the car, then the behavior of the driver in the refueling process can reduce the service lifetime of the tank by 78% and have significant negative effects.
4. It is suggested that the designers of CNG tanks should consider more about using the standards, and sometimes some recommendations are needed to add to the standards and even update the old versions.
5. The most important advice to car drivers is to refuel when the CNG tank is completely empty.

## Figures and Tables

**Table 1:** Characteristics of cyclic loading in different modes investigated to find the effect of driver’s behavior in refueling on the service life of the tank.

Name	Minimum Load (bar)	Maximum Load (bar)	Mean Value (bar)	Loading Ratio
SL	20	200	110	0.1
DB-1	38	200	119	0.19
DB-2	56	200	128	0.28
DB-3	74	200	137	0.37
DB-4	92	200	146	0.46

**Table 2:** Service life of the composite CNG tank via the mileage of the vehicle based on the conditions mentioned in this research.

Loading Mode	SL	DB-1	DB-2	DB-3	DB-4
Service life of type-IV CNG tank (km)	4,340,000	3,566,000	2,703,000	1,824,600	934,000
Reduction compared to the standard mode (%)	00.00	17.83	37.72	57.96	78.48

## Data Availability

The data that support the findings of this study are available from the corresponding author upon reasonable request.
